# Fluctuation in Pupil Size and Spontaneous Blinks Reflect Story Transportation

**DOI:** 10.16910/jemr.13.3.6

**Published:** 2020-06-01

**Authors:** Johanna K. Kaakinen, Jaana Simola

**Affiliations:** University of Turku, Finland; University of Helsinki, Finland

**Keywords:** Eye tracking, pupillometry, eye blinks, literary texts, horror, emotion, immersion, transportation

## Abstract

Thirty-nine participants listened to 28 neutral and horror excerpts of Stephen King short stories while constantly tracking their emotional arousal. Pupil size was measured with an Eyelink 1000+, and participants rated valence and transportation after each story. In addition to computing mean pupil size across 1-sec intervals, we extracted blink count and used detrended fluctuation analysis (DFA) to obtain the scaling exponents of long-range temporal correlations (LRTCs) in pupil size time-series. Pupil size was expected to be sensitive also to emotional arousal, whereas blink count and LRTC’s were expected to reflect cognitive engagement. The results showed that self-reported arousal increased, pupil size was overall greater, and the decreasing slope of pupil size was flatter for horror than for neutral stories. Horror stories induced higher transportation than neutral stories. High transportation was associated with a steeper increase in self-reported arousal across time, stronger LRTCs in pupil size fluctuations, and lower blink count. These results indicate that pupil size reflects emotional arousal induced by the text content, while LRTCs and blink count are sensitive to cognitive engagement associated with transportation, irrespective of the text type. The study demonstrates the utility of pupillometric measures and blink count to study literature reception.

## Introduction

Literary texts have the power to induce rich emotional experiences, either by their content (e.g., the thrill and suspense felt during reading of Stephen King novels) or form (e.g., the awe produced by the skillful use of language) (1, 2, 3). Sometimes a literary text can be so engaging that we “get lost in the story world” – a concept that has been coined as immersion, transportation, or absorption (4, 5, 6). When immersed in the story world, “… all mental systems and capacities become focused on events occurring in the narrative”(5). We might feel suspense and eagerly expect what will happen next, form vivid mental imagery of the scenery and the locations described in the story, and get emotionally involved and touched by the emotions of the story characters (7).

But what exactly are the cognitive and affective mechanisms underpinning an immersive literary experience? The Neuro-Cognitive Poetics Model (NCPM) recently proposed by Jacobs (8) addresses the question of emotional processes during reception of literary art. First of all, the NCPM posits that the same neural circuitry that is responsible for emotional reactions in the real world is also involved in creating a literary experience. This assumption is based on brain imaging studies showing that emotional words and passages activate brain areas that are responsible for processing of other types of emotionally significant stimuli (e.g., 9, 10, 11). Another basic assumption of the model is that strongly emotional text content is more likely to induce emotions in the reader and create feelings of empathy towards the story characters, which enhances immersion. This assumption is supported by empirical findings showing  that high immersion in emotionally charged segments of a novel (in this case, Harry Potter series) is related to activation of brain regions associated with experiencing affect and empathy (e.g., 12). Finally, the NCPM posits that high transportation or immersion should increase processing fluency, which should be reflected in, for example reading, as shorter eye fixation times.

The dimensional emotion theories, such as the circumplex model of emotion posit that emotions can be described in two dimensions: arousal and valence (13). Arousal refers to the intensity of the emotional activation, whereas valence describes how pleasant or unpleasant the experience is. As an example of emotions induced by literary texts, consider the horror story *1922* written by Stephen King. It contains a detailed description of how a father and a son sneak into the parent’s bedroom at night, attack the mother of the family, slice her throat and brutally mutilate her. This sort of a text excerpt can be expected to induce a high arousal, negative emotion in the recipient, a feeling that intensifies as the terrible events unfold. On the other hand, the same story includes a description of a mundane weather conversation between family members while they are sitting on their front porch, watching a sunset. This text excerpt is likely to induce a very different reaction than the one described before: lower arousal and a neutral or even slightly positive emotion. 

Previous research shows that emotional responses influence attentional and memory processes (e.g., 14). Especially arousal is crucial in how attentional resources are allocated, and highly arousing stimuli tend to capture and maintain attention (see e.g., 15, 16). This means that there is a strong link between emotional arousal and cognitive engagement (i.e., concentration of attentional and memory processes), and that highly arousing stimuli like horror stories should capture attention and be more cognitively engaging than less arousing materials. What remains an open question is the relationship between cognitive engagement and experiences of transportation. If higher arousal induces higher cognitive engagement, then one could argue that it is also more likely to induce higher story transportation. This reasoning is in line with the NCPM (8), which posits that different text-, context- and recipient-related features determine the emotional responses to text. For example, higher immersion to an emotional story would induce higher empathy towards the story characters and more vivid emotional experiences. 

Despite the growing interest in utilizing empirical methods such as eye tracking to study literary experience (see e.g., 17, 18, 19), very little is still known about the interplay of emotional and cognitive processes during literary text reception. The goal of the present study was twofold. First, we were interested in how emotionally arousing  text content (specifically, horror) influences the emotional and cognitive processes occurring during literary text reception and the experiences of transportation. Second, we were interested in how transportation to the story world is reflected in the measures of emotional and cognitive engagement. In order to answer these questions, we combined subjective reports of emotion (arousal and valence) and transportation with measures derived from eye tracking (pupil size and eye blinks) collected while participants listened to excerpts of literary texts from the horror genre.

### Measuring arousal and cognitive engagement

An emotional response involves a subjective experience, physiology and behavior, and a combination of different methods is needed to describe the interplay of these different facets of emotion (20). Subjective experience can be measured with self-reports, such as the self-assessment manikin (SAM) scales introduced by Bradley and Lang (21). The SAM is a pictorial scale consisting of images denoting different dimensions of emotional experience. The valence scale includes manikins expressing a continuum from an unpleasant to a very pleasant feeling, and the arousal scale contains images representing a continuum from a very calm to an extremely agitated “explosive” state. Participant’s task is to indicate which manikin corresponds to their emotional experience. The SAM is an easy and quick way to assess the subjective emotional experience in different contexts (21). In the present study, we used the arousal scale to track continuous changes in experienced arousal during listening of the stories. The valence scale was used after each text as a manipulation check. 

Changes in arousal are controlled by activation of the autonomic nervous system (ANS), typically indexed by physiological measures such as pupil size, galvanic skin response, or heart rate (22, 23). In the present study we were interested in pupil size, which is controlled by two muscles: the dilator and the sphincter (24), which in turn are influenced by activity in the two parts of the ANS, the sympathetic and parasympathetic systems (23). Pupil size is further associated with the locus coeruleus – norephinephrine (LC–NE) system, which is a major neurotransmitter system modulating general arousal and attention (25). Prior research indicates that the pupil dilates in response to the interest value of the pictures (26), and that emotionally arousing stimuli, such as emotional pictures (22) and sounds (27) induce pupil dilation when compared to emotionally neutral stimuli.  

Pupil size is not only sensitive to emotional arousal but it also reflects the cognitive demands of a task (28). For example, the size of the pupil increases with the difficulty of the problem in a mental multiplication task (29) or with the number of items required for recall in a short-term memory task (30). These early studies indicate that the pupil dilates relative to baseline levels due to increases in cognitive processing load. More recent studies also show that pupil size can be modulated by attention (31, 32, 33) and working memory even in the absence of visual stimulus presentation or anticipation (34). These results propose that pupil dilation can be used as an indirect marker of cognitive load and possibly also cognitive engagement during task performance. 

Studies using pupillometry typically report task evoked pupillary responses (TERPs) under a variety of conditions and disregard dynamics in continuous pupillary signals. Only a few earlier studies have analysed the nonlinear dynamics of the pupil signal (35, 36). An interesting application of pupillometry is to analyze the scale-free dynamics of pupil size fluctuations, which reflect the brain state underlying cognitive performance. Human cognitive and behavioral performance (37, 38, 39) as well as neuronal activity (40, 41) is known to fluctuate in time scales from seconds to tens or hundreds of seconds such that successive observations show similar outcomes more likely than expected by chance. These autocorrelations exhibit power-law distributed long-range temporal correlations (LRTCs). Power-law scaling behavior and LRTCs suggest that the underlying neural system operates near a critical state (40, 42). Operating near criticality provides an optimal processing capacity and flexibility in reconfiguration among possible states (42, 43, 44). Strong LRTCs have been shown to parallel optimal cognitive flexibility, indicating a functionally advantageous state (39). In the present study, we used LRTC’s to examine differences between emotionally arousing and neutral texts, and to explore the cognitive underpinnings of story transportation. 

In addition to pupil size, eye movement recordings can be used to compute the number and frequency of spontaneous eye blinks, which have been found to reflect the cognitive demands of the task (see 45). For example, average blink rate during conversation is 26 blinks/min, whereas during reading it is only 4.5 blinks/min (46). Previous research suggests that blinking is inhibited when the task requires high cognitive engagement or attention, especially in the visual domain but also in other modalities (45, 46, 47). On the other hand, mind-wandering or zoning out episodes are characterized by increased blinking, indicating that there is a relationship between blinking and (dis)engagement of attention (48). Moreover, a brain imaging study showed that spontaneous blinks are associated with momentary inhibition of the dorsal attentional network, which controls the allocation of attention, and with activation of default mode network (DMN) (49), which has been implicated in mind-wandering or zoning-out (50). In a recent study on viewing of emotional film clips (51), blink rate was negatively correlated with self-reported interest: higher the interest, lower the blink rate. Based on these previous findings, it can be assumed that higher cognitive engagement with emotional stimuli reduces the frequency of blinks.

### Arousal and transportation during literary text reception

Only a few previous studies on literary text reception have utilized measures that tap directly into the ANS activation. In a study by Wallentin et al. (52), participants listened to a 21-minute recording of the story *Ugly Duckling* by H.C. Andersen and self-reported their emotional arousal or valence for each line of the transcribed text. Another group of participants then listened to the same recording while their heart rate variability (HRV) was measured. The peaks in arousal ratings for different text segments correlated with observed changes in HRV, indicating that arousing story events triggered ANS activation in the story recipients. 

In a study on story transportation, Riese, Bauer, Lauer and Schact (53) used pupillometry to examine emotional reactions during listening of sections of *The Rider on the White Horse* by Theodor Storm and *Effi Briest* by Theodor Fontane. They collected suspense ratings following the procedure of Wallentin et al. (52) to get a continuous measure of suspense across the story. Another group of participants then listened to the stories and rated them for different aspects of transportation, including emotional involvement. The results showed that the Rider on the White Horse was more suspenseful and induced higher emotional involvement than Effi Briest. The results of the pupil size analyses showed that in the end of the text sections there was a weak correlation (*r*=.25 for The Rider of the White Horse, and *r*=.21 for Effi Briest) between the continuous suspense rating and pupil size. These results suggest that suspenseful segments of literary stories induce higher ANS activation, as reflected in pupil size, which correlates with higher emotional involvement with the text.

### Overview of the present study

The purpose of the present study was twofold. First, we examined the emotional responses and cognitive engagement during listening of emotional (horror) and neutral text excerpts. Second, we were interested in how transportation to the story world is reflected in the measures of emotional arousal and cognitive engagement. Participants listened to short stories containing either negatively valenced horror content or neutral excerpts taken from the same stories, while constantly tracking their arousal level. After each text, participants responded to a valence scale and a short form of the transportation scale (54). Eye tracking was used to measure pupil size and to detect blinks during story presentation. Mean pupil size across the story presentation was used as a measure of ANS activation, and two measures of cognitive engagement were employed: long-range temporal correlations (LRTCs) in pupil size fluctuations, and blink count. 

Based on the assumptions of the NCPM (8), we predicted that literary descriptions of emotionally provoking events produce an emotional arousal response, as reflected in self-reports and pupil size. Moreover, emotional texts were expected to induce higher transportation, which should be associated with higher cognitive engagement (stronger LRTCs in the pupil data and reduced blink count) during story listening.

## Methods

### Participants

Fourty-four University of Turku students participated in the experiment for partial course credit or a movie ticket. All participants signed an informed consent before the experiment. Participants were native speakers of Finnish (the language used in the materials) and reported no neurological disorders, substance use, or medication that would influence the central nervous system. Due to unexpected software crashes during recordings, data for five participants was lost, and the final dataset contained data from 39 participants (5 males), whose mean age was 23.36 years (SD = 3.62years).

### Apparatus

Pupil size was recorded with a desktop-mounted Eyelink 1000+ (SR Research Ltd.) eye tracker using 500Hz sampling rate in the remote mode (for technical specifications of the eye tracker, see 55). Visual stimuli (i.e., arousal scale during story listening, and valence scale and transportation questions after listening, see below) were presented centrally on a 24-inch BenQ XL2420Z LCD screen using 1920x1080 resolution and 100Hz refresh rate.

### Materials

Text materials consisted of 29 excerpts of Stephen King short stories translated to Finnish. In order to control for effects of emotional prosodic cues, audio files of the stories were created with text-to-speech software available in Microsoft Word, using female voice at normal reading speed. The excerpts were selected from the short stories written by Stephen King on the basis of their content: 12 texts included emotionally provoking (horror) content and 17 were neutral. One of the neutral texts was used in a practice trial in the beginning of the experiment. The original stories were edited to ensure that the excerpts were of comparable length while they would still describe a clear event or present a comprehensible part of a dialogue. The horror stories were on average 969 characters (SD=82.33) and 149 words long (SD=13.24), and the neutral texts were on average 939 characters (SD=65.31) and 145 words long (SD=12.19). The mean duration of the audio files was 82.39 s (SD=6.80) for the horror, and 81.83 s (SD=5.44) for the neutral stories, respectively.

The self-assessment mannikins (SAM, 21) were used to assess arousal and valence. SAM is a pictorial scale consisting of nine images presenting a continuum from extremely calm to extremely aroused state in the arousal scale, and from extremely negative to positive mood in the valence scale. During presentation of the audio files, the arousal scale was presented on the computer screen and participants were asked to constantly track their arousal by pointing a red arrowhead controlled with mouse on the image that corresponded to their emotional state. When the text finished, the valence rating scale was presented on the screen, and participants were instructed to click on the image that matched their emotional state. The x-coordinate of the mouse position/click on the screen was used as the measure of arousal and valence.

Narrative transportation was measured after each text with transportation scale short form (TS-SF) (54). The scale consists of six items measuring general, cognitive, emotional and imaginative facets of narrative transportation (e.g., “I was mentally involved in the narrative while reading it”), and participants respond to statements on a scale from 1 to 7 (1=not at all, 7=very much). The original scale includes two items measuring the vividness of mental imagery separately for each of the story characters (e.g., “While reading the narrative I had a vivid image of Katie”). For practical reasons, we included only one general item on vividness of imagery (“While reading the narrative I had a vivid image of the story characters”), and thus the scale in the present study consisted of five items. Items were presented one at a time on the computer screen and participants responded with keypresses on the numeric keyboard. Mean across the responses was computed and used as a measure of transportation.

### Procedure

The study protocol was approved by the ethics committee of the University of Turku. Participants signed the informed consent form upon arrival to the laboratory. Participants were then given headphones and written instructions were presented on a computer screen, informing participants that they were going to listen to short stories, some of which may contain emotional content. They were told that we were interested in how they felt during story listening, and that their pupil size will be measured with an eye tracker. Participants were instructed to track their arousal during listening of the texts, and to respond to questions concerning their experience after each text.

The specific instructions for the arousal tracking stated that the task was to evaluate how aroused the participant felt during listening of the stories. The nine SAM arousal images and a clearly visible red arrowhead denoting the location of the mouse cursor were presented on the screen below the written instructions. In the instructions, participants were told that they can control the location of the red arrowhead with a mouse and that the arrowhead should be placed on the image that matches their arousal at a given moment. They were then instructed that images on the left represent a completely calm feeling whereas images on the right represent an extremely aroused state of mind. Finally, the instructions emphasized that the task is to use the red arrowhead to track possible changes in felt arousal during story presentation. 

After the arousal tracking instructions, instructions for responding to the valence rating task were presented. The nine SAM valence images were presented on the screen with a red arrowhead denoting mouse cursor, and participants were told that after story presentation they should indicate the valence of their emotional state induced by the text. Participants were told to place the red arrowhead on the image that matches their emotion and to click on the mouse as a response. They were then instructed that images on the left represent a very negative emotion, images on the right an extremely positive emotion, and that the image in the middle represents a completely neutral emotion. Finally, participants were told that after the valence rating they will answer some questions of how they experienced the text, and that responses are given with the numeric keys on the keyboard.   

After the instructions, the eye tracker was calibrated, and a neutral practice text was presented before the actual experiment. During practice trial participants rated their arousal, and responded to the valence and transportation scale after the text as in the actual experiment to familiarize them with the procedure. The 28 experimental texts were presented via headphones in a randomized order. In the beginning of each text, the SAM arousal scale was presented on the screen, and participants were to click on the image indicating their current state of mind. After a 1 second delay, the audio file started. After each text, the SAM valence rating and the TS-SF items were presented on the screen. The whole session lasted for about 1 hour.

## Results

### Data preparation

Mouse coordinates recorded during the listening task were used as a measure of arousal. Two participants had misunderstood the instructions and were excluded from the analyses, and the final dataset for self-reported arousal contained data from 37 participants. Observations that did not fall within the screen area in which the SAM image was presented (x:540-1380pxls, y:470-610pxls) were excluded (3.25% of the original arousal rating data) and coded as missing data. The x-coordinate was taken as the value of arousal, and a mean per each second was computed for each text and each participant.

From the pupil size data, blinks were identified using the Eyelink parser blink detection algorithm (SR Research, Ltd. Ontario, Canada), which identifies blinks as periods of loss in pupil data. Saccades were also identified using Eyelinks’s algorithm. Mean pupil size per each second of the audio file was then computed to examine the overall changes in pupil size during listening of the texts. Blink count per text was based on the number of consecutive samples marked as blinks by the Eyelink algorithm, excluding sequences that were shorter than 100ms or longer than 500ms. These data were available from 39 participants. 

In order to examine the dynamics of the pupil size fluctuations, raw pupil size data were preprocessed using custom scripts and third party toolboxes in MATLAB version 9.6 based on criteria previously used by Fink et al. (56). Samples consisting of blinks or saccades were set to NaN, as was any sample that was 4 arbitrary units greater than the preceding sample. A window of 25 samples (50ms) was used around all NaN events to remove edge artifacts. Missing pupil data were linearly interpolated. Trials requiring 50% or more interpolation were discarded from further analysis (see 57), which equated to 39% of the data (all data of one participant had to be excluded on this basis). Finally, the pupil data were downsampled to 50 Hz. To examine dynamics of scale-free pupil size fluctuations, we estimated their long-range temporal correlations (LRTCs) using power-law exponent from detrended fluctuations analysis (DFA) (58, 59). DFA was applied to the time series of preprocessed pupil size data in two stages. First, time series X(k) was normalized to zero mean and the cumulative sum of the signal was computed. The integrated signals from each trial were then segmented into multiple time windows Δt from 10 to 50–80 s (length multiplier 1.1). The maximum window length varied from trial to trial depending on the audio file length, which ranged from 71.10 to 92.80 s with a mean of 83.35 s (SD=5.82). Second, each segment of integrated data was locally fitted to a linear function y_Δt_(k) and the mean-squared residual F(Δt) was computed. The power law scaling exponent β was defined as the slope of linear regression of the function F(Δt) plotted in log–log coordinates, estimated using a least-squares algorithm. The final dataset of the power law scaling exponents contained data from 38 participants.

Valence rating was the x-coordinate of the mouse click recorded during the valence rating task after each text. A mean score for transportation was computed on the basis of the participant responses (on a scale 1-7) to the TS-SF items. Valence and transportation ratings were available from 39 participants.

### Statistical analyses

The data were analyzed with (generalized) linear mixed models using the lme4 package (60) for R statistical software (61). Blink count was analyzed with a generalized linear mixed model using Poisson distribution. All other measures were analyzed with linear mixed models (lmm’s) using REML estimation. Results for the SAM scale measures (arousal and valence) were double-checked with cumulative linked mixed models for measures transformed from the raw x-coordinates to ordinal scale (1-9); as the results were similar as in the lmm’s, the analyses of the original measures are reported. Two sets of analyses were conducted: 1) Comparisons between horror and neutral texts, and 2) analyses to examine the association between transportation and the dependent variables (DVs). Separate models for each DV were computed.

The models comparing arousal and mean pupil size (DV’s) during listening of horror and neutral texts were of the form:

DV ~ Text type * Time + (Text type * Time | Participant) + (Time | Item)

Text type (coded using sum contrast), time (centered), and their interaction term were included as fixed effects. Random intercepts for participants and items and the random slopes for all fixed effects, including the interaction term at the level of participants, were included in the random part of the models.

There was only one observation per text for each participant for valence, blink count, and power law scaling exponent (β), and thus the models for comparisons between horror and neutral texts for these DV’s were of the form:

DV ~ Text type + (Text type | Participant) + (1 | Item)

The second set of analyses examined how transportation was reflected in the dependent measures during and after story listening. The models for arousal and mean pupil size (DV’s) were of the form:

DV~ Transportation * Time + (Transportation * Time | Participant) + (Transportation * Time | Item)

In the models, transportation rating (centered), time (centered), and their interaction term were included as fixed effects. Random intercepts for participants and items and the random slopes for all fixed effects, including the interaction terms, were initially included in the random part of the models. However, for pupil size the random slope for the interaction term at the item level was dropped from the final model due to overidentification.

For blink count and power law scaling exponent (β), the initial models were of the form:

DV ~ Transportation + (Transportation | Participant) + (Transportation | Item)

For β the full model proved singular, and the model was trimmed by removing the random effect with the smallest variance component until an acceptable model was reached. The final model included only a random intercept and slope of transportation at the participant level.

### Differences between horror and neutral texts

**Figure 1.**
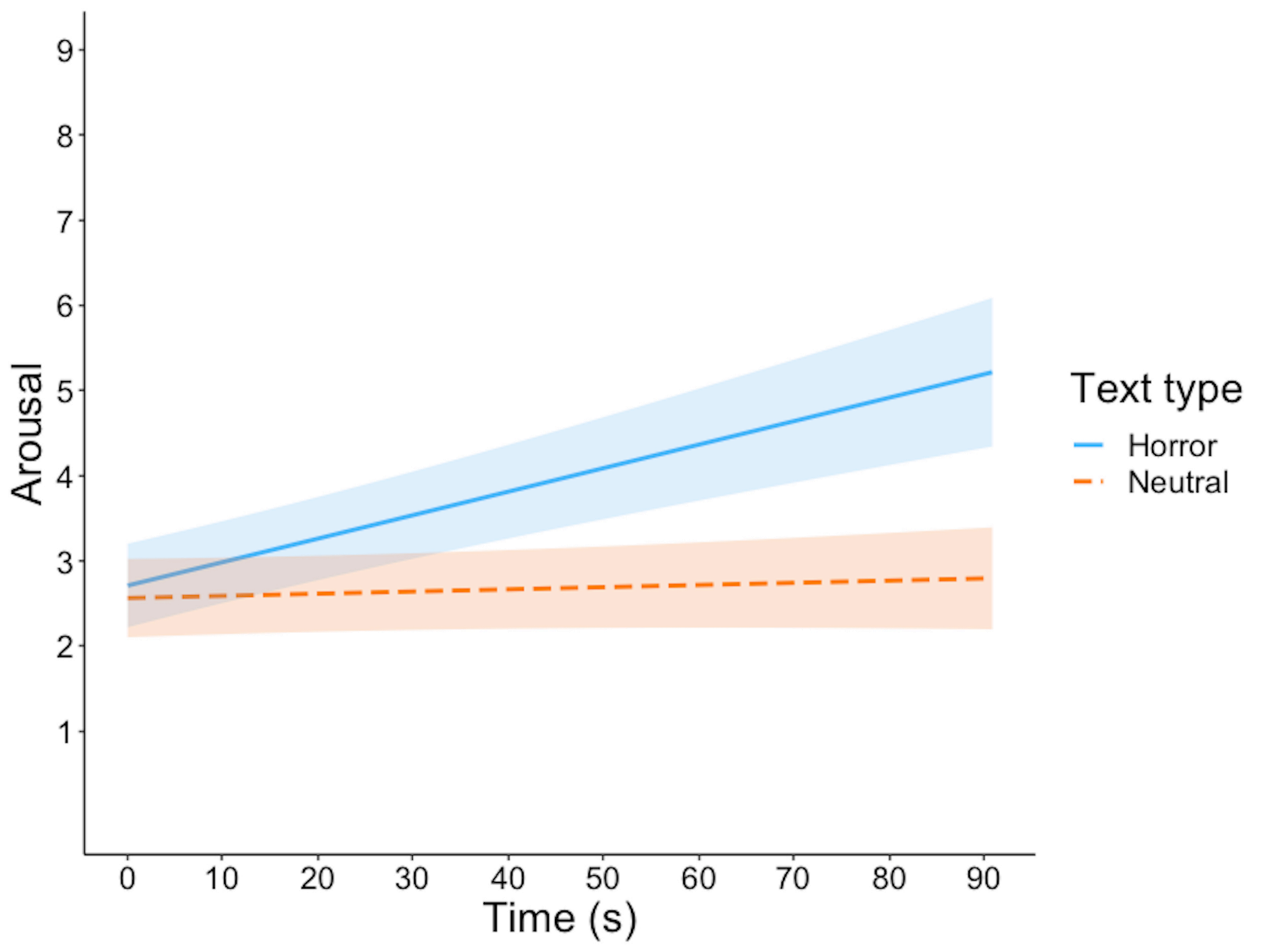
Model estimates for self-reported arousal. The scale on the y-axis is presented in discrete values corresponding to the SAM scale images (1 = calm, 9 = extremely agitated) for the sake of clarity. Shaded areas represent 95% confidence intervals.

We first examined the impact of text type on measures recorded during the course of listening. Horror texts induced overall higher self-reported arousal than neutral texts, b = 54.15, 95%CI = [32.63,75.67], SE = 10.98, t = 4.93. There was also an overall increase in reported arousal across time, b = 33.92, 95%CI = [22.80,45.05], SE = 5.68, t = 5.98. More importantly, there was an interaction between elapsed time and text type, b = 28.17, 95%CI = [15.39,40.94], SE = 6.52, t = 4.32, indicating that the increase in arousal depended on text type. The nature of this interaction is evident in Figure 1: there is a steep rise in self-reported arousal during listening of horror texts whereas there is practically no change in arousal during listening of neutral texts.

**Figure 2.**
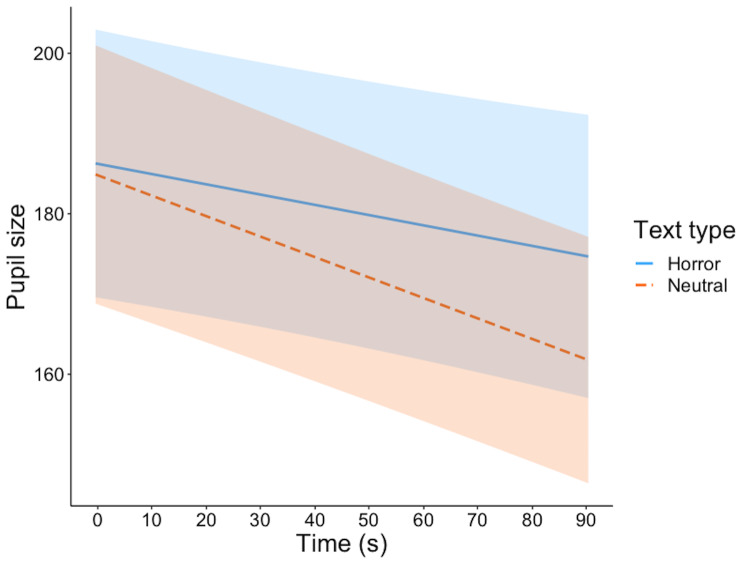
Model estimates for pupil size. Shaded areas represent 95% confidence intervals.

Mean pupil size was overall larger during listening of horror than neutral texts, b = 3.29, 95%CI = [1.20,5.37], SE = 1.06, t = 3.09. The pupil size decreased during the presentation of the text, b = -4.61, 95%CI = [-6.27,-2.95], SE = .85, t = -5.45, but more importantly, there was an interaction between time and text type, b = 1.53, 95%CI = [.35,2.71], SE = .60, t = 2.55. As can be seen in Figure 2, this interaction reflects that the decrease of pupil size during listening is steeper for neutral than for horror stories.

The analysis of the scaling exponents, β, obtained from the detrended fluctuation analysis (DFA), did not reveal differences in the LRTCs of pupil size fluctuations between horror (M = .94, SD = .25) vs. neutral texts (M = .93, SD = .14) (average goodness of fit, r^2^ = .85, SD = .14 and r^2^ = .87, SD = .07, respectively), t < 1. There were no differences in blink count during listening of horror (M = 40.15, SD = 25.29) and neutral texts (M = 40.76, SD = 22.53), z < 1.

**Figure 3.**
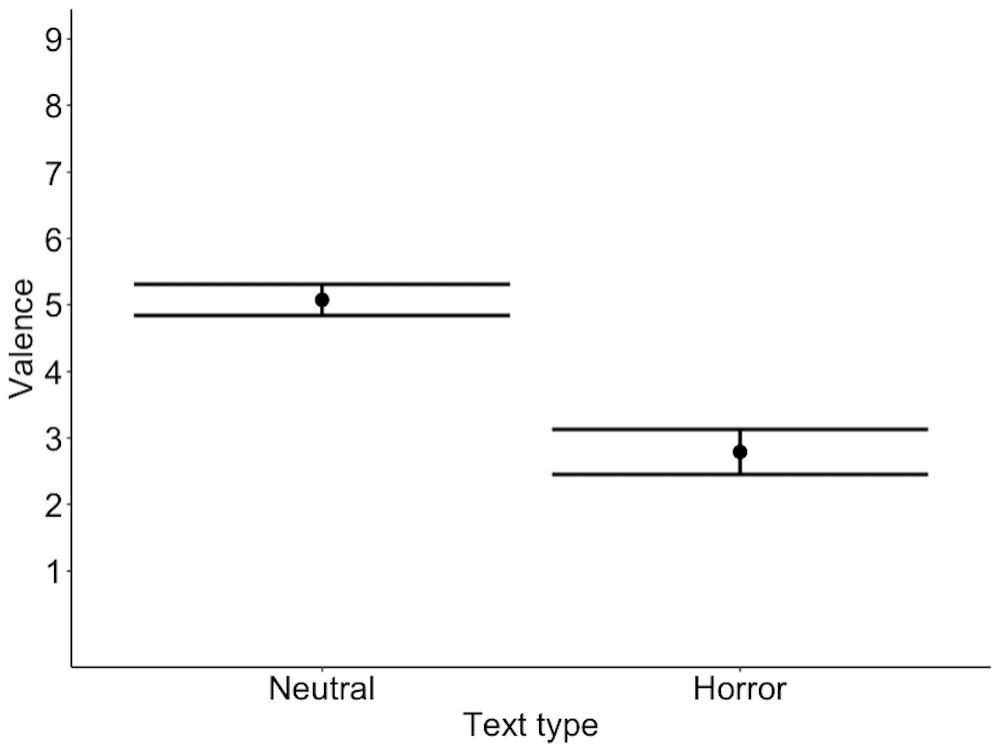
Model estimates for valence ratings. The scale on the y-axis is presented in discrete values corresponding to the SAM scale images (1 = extremely unpleasant, 9 = extremely pleasant) for the sake of clarity. Error bars represent 95% confidence intervals.

**Figure 4.**
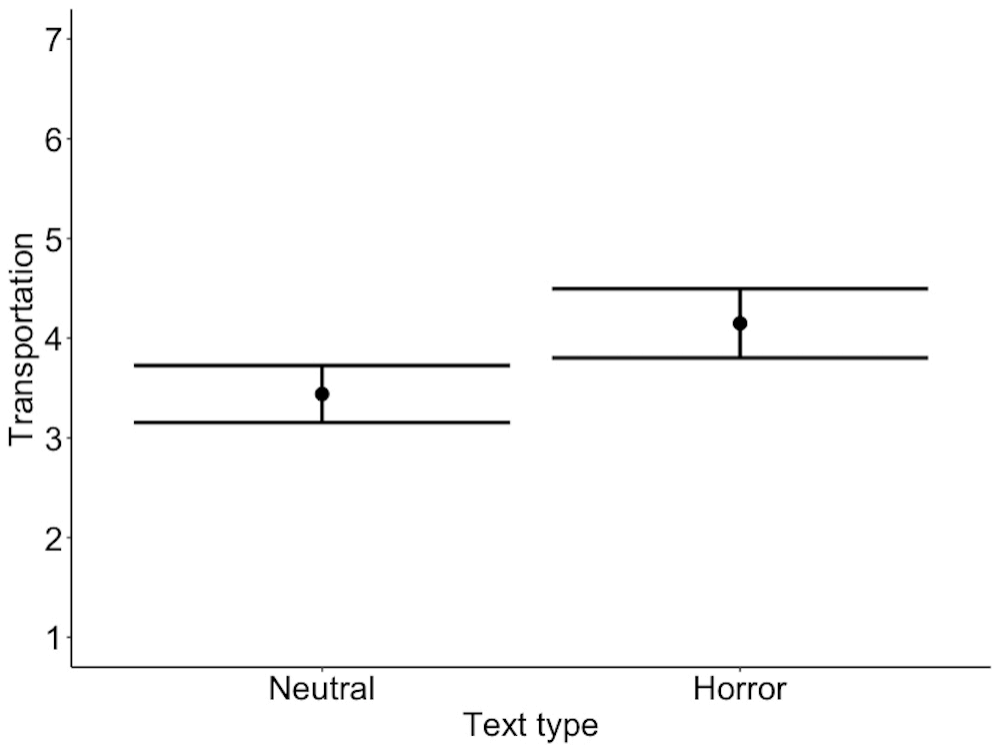
Model estimates for transportation ratings. Error bars represent 95% confidence intervals.

Finally, we examined the ratings given after listening to each text. The texts differed in emotional valence: neutral texts were rated as more positive (M = 1013.81, SD = 54.18) than horror texts (M = 800.32, SD = 86.84), b = 106.75, 95%CI = [88.26,125.24], SE = 9.43, t = 11.32, confirming that our manipulation worked (see Figure 3). Neutral texts were rated as neutral, whereas horror texts were rated as unpleasant. Moreover, horror texts induced higher transportation (M = 4.15, SD = .90) than neutral texts (M = 3.44, SD = .73 ), b = .35, 95%CI = [.18,.53], SE = .09, t = 4.05 (see Figure 4).

### Effects of transportation

**Figure 5.**
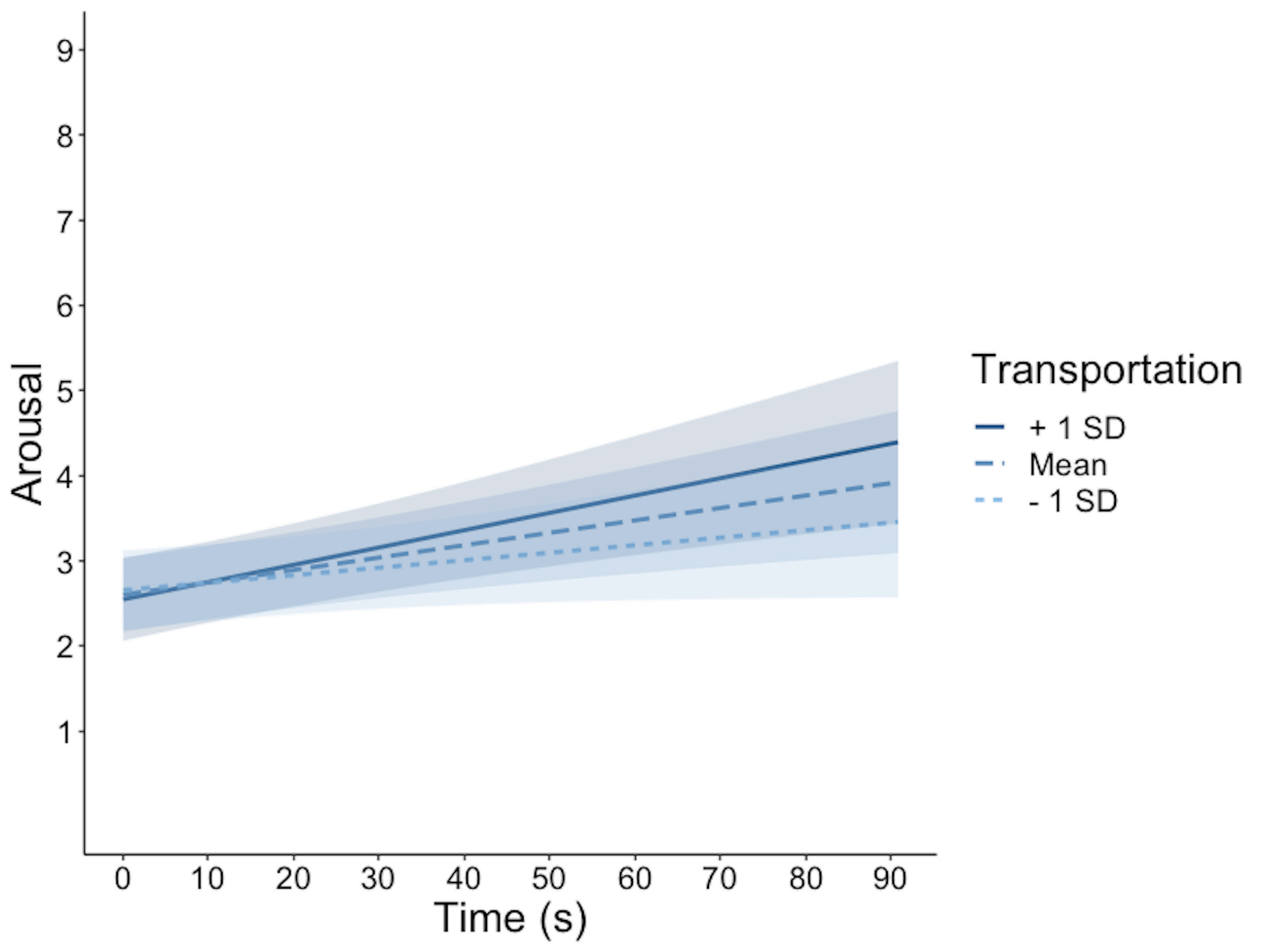
Model estimates for self-reported arousal. The scale on the y-axis is presented in discrete values corresponding to the SAM scale images (1 = calm, 9 = extremely agitated) for the sake of clarity. The different lines represent model estimates at transportation scores 1SD below the mean, at the mean, and at 1SD above the mean. Shaded areas represent 95% confidence intervals.

As was already indicated in the text type analyses, self-reported arousal linearly increased across time, *b* = 32.79, *95%CI* = [13.61,51.97], *SE* = 9.79, *t* = 3.35. Arousal was also slightly overall higher if the experienced transportation was high, *b* = 16.72, *95%CI* = [-.21,33.64], *SE* = 8.63, *t* = 1.94. More importantly, there was an interaction between time and transportation, indicating that the increase in arousal depended on the level of transportation, *b* = 12.95, *95%CI* = [1.31,24.59], *SE* = 5.94, *t* = 2.18. As is evident in Figure 5, higher the transportation, steeper the slope in experienced arousal.

**Figure 6.**
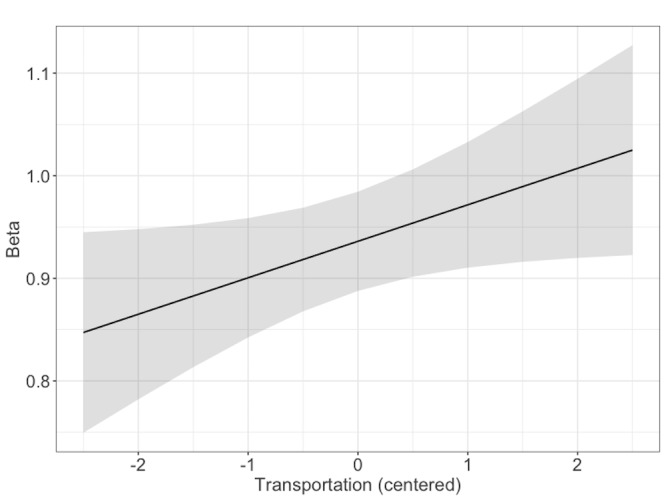
Model estimates for LRTC scaling exponents (β) from the DFA of pupil size fluctuations. The shaded area represents 95% confidence interval.

As was already seen in the text type analyses, mean pupil size decreased across time, b = -5.07, 95%CI = [-6.92,-3.21], SE = .95, t = -5.36. However, there was no evidence for an overall effect of transportation or interaction, t’s < 1. Instead, transportation was a significant predictor of the LRTC scaling exponents, β, from the DFA, b = .04, 95%CI = [.0006,.07], SE = .02, t = 1.99, indicating that higher transportation was associated with greater LRTCs in pupil size during story listening (see Figure 6).

**Figure 7.**
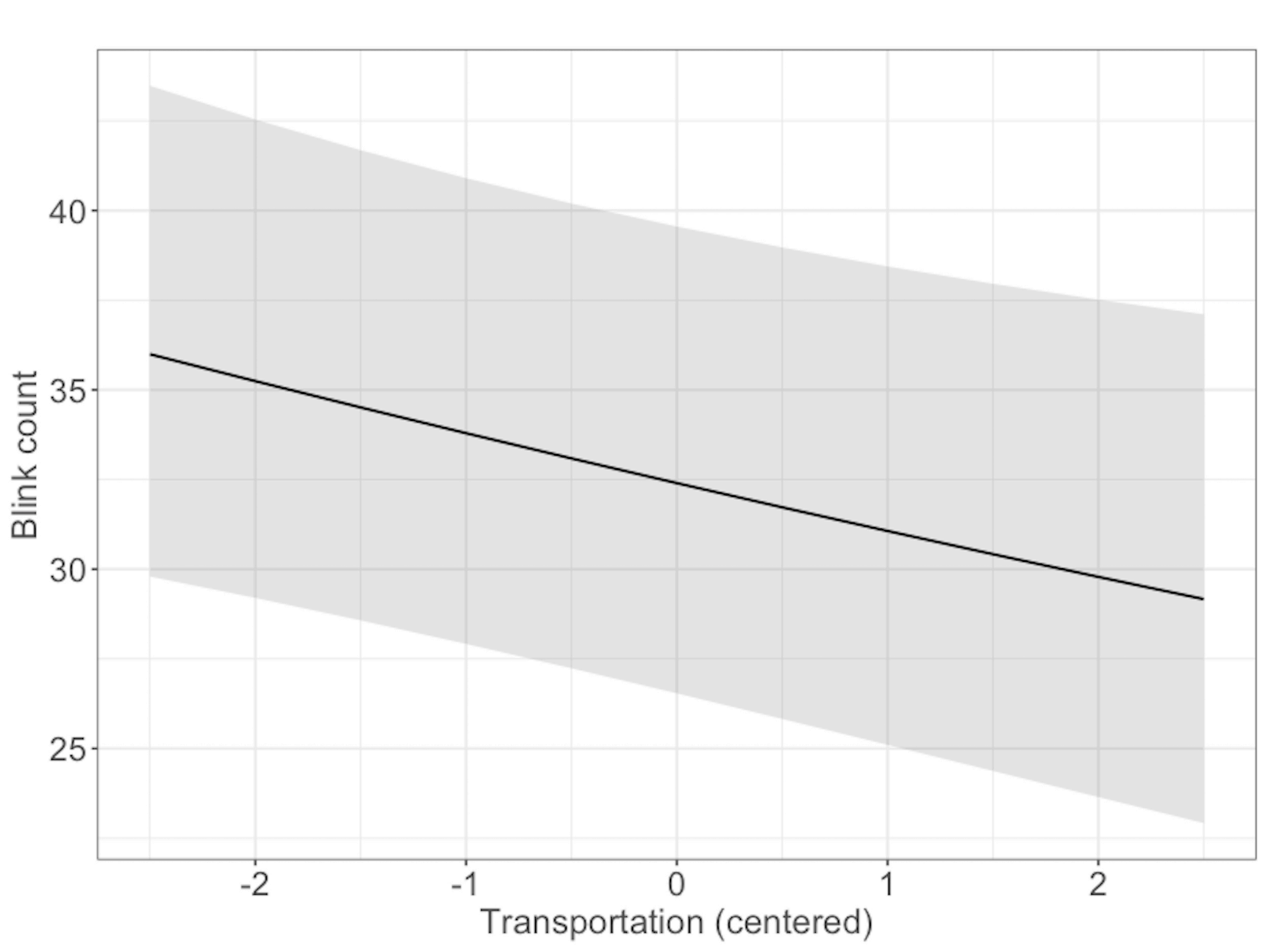
Model estimates for blink count. The shaded area represents 95% confidence interval.

Finally, the analysis of the blink count (see Figure 7) indicated that high transportation was related to reduced blinking, b = -.04, 95%CI = [-.08,-.008], SE = .02, z = -2.46.

## Discussion

The goal of the present study was twofold. First of all, we examined differences between horror and neutral texts in emotional and cognitive processing by collecting subjective reports of experienced arousal, valence and transportation, as well as by analysing changes in the mean pupil size, LRTCs of pupil size fluctuations and blink count during story listening. Second, we were interested in the emotional and cognitive underpinnings of transportation, and examined the associations between experienced transportation and arousal, pupil size, LRTCs, and blink count.

The comparisons between horror and neutral texts showed that emotional arousal steadily increased during exposure to horror stories, as indicated by both the self-reported arousal measure and the mean pupil size, whereas there was practically no change (in self-reported arousal) and even a decline in arousal for neutral texts as indexed by the steeper decrease of pupil size over time. The overall decrease in pupil size observed across the listening task indicates that there is an orienting response in the beginning of text, which wears off across time (for time-on-task effects, see e.g., 33). Our interpretation of the smaller decrease in pupil size for the horror texts than for the neutral texts is that horror stories induce more ANS activation (i.e., arousal) than neutral texts. The present results are in line with a previous study on literary reception by Wallentin et al. (52), which demonstrated that segments of Ugly Duckling that were rated as highly arousing also induced higher ANS activation, as measured by HRV. Moreover, the present results indicated that in addition to inducing higher arousal, horror texts also produced higher transportation than neutral texts. These findings are in line with the NCPM (8) by demonstrating that horror texts induce a stronger emotional arousal reaction, as well as higher transportation, than neutral texts.

As pupil size is sensitive to both emotional arousal and cognitive effort, the present results could also be interpreted as horror texts inducing higher cognitive load than neutral texts. However, the lack of text type effects on blink count suggests that the effects on the mean pupil size here are more likely to reflect emotional arousal than cognitive effort. Thus, we obtained little evidence showing that horror texts would increase overall cognitive engagement with the text.

The analyses of the transportation effects indicated that higher transportation was associated with a steeper increase in self-reported arousal during story presentation, stronger LRTCs, and reduced blink count. These results imply that transportation is characterized by changes in experienced arousal and higher cognitive engagement. The lack of transportation effects on the mean pupil size suggest that transportation might not be associated with emotional ANS activation – rather, transportation seems to invite more efficient or fluent cognitive processing, as indicated by the LRTCs and blink count. In line with previous research (45, 46, 47), the reduced blink count during higher transportation specifically implicated the relationship between cognitive engagement and transportation. Our results thus suggest that the mean pupil size and LRTCs of pupil size fluctuations tap into different processes during story listening, as the mean pupil size over time differentiated between the emotional valence of the stories while LRTCs correlated with the story transportation. Similarly, Simola et al. (39) found that LRTCs in response time (RT) time series were uncorrelated with the mean and SD of RTs in a Go/NoGo task (see also 35). These findings are generally in line with the NCPM (8), which posits that high immersion is characterized by fluent processing.

In a previous study by Riese and colleagues (53) pupil size correlated with ratings of suspense, which is one of the core aspects of transportation. This is somewhat in disagreement with the present results, as we did not find evidence for an association between pupil size and transportation. However, the observed correlations in Riese et al. were weak, and only appeared towards the end of the texts. The present study differs from the Riese et al. study in that we utilized a general measure of transportation, containing items on cognitive, emotional and imaginative facets of transportation, instead of looking at suspense specifically. Another difference is that we used multiple shorter texts (145-149 words long), whereas Riese et al. used two longer sections (1362-1418 words long) taken from novels, one from a suspenseful and another from a neutral novel. One possible explanation for the different results is that suspense is more specifically related to physiological arousal than a “general” experience of transportation, which could be why Riese et al. observed a correlation with pupil size and we did not. It should also be noted that Riese et al. only reported correlations between suspense ratings and pupil size and did not report whether there was an overall difference between the suspenseful and neutral texts, or whether the pupil size changed differently during listening of the texts, leaving open the question of whether text’s emotional tone plays any role in the build-up of arousal during literary reception.

The present study demonstrates that pupillometric variables can be used to study literary experiences, and that different measures tap into emotional arousal and transportation. Pupil size correlated with emotional arousal triggered by text content, whereas the LRTCs of pupil size fluctuations and blink count correlated with transportation, implying that transportation is characterized by processing fluency and/or higher cognitive engagement. Stronger LRTCs have previously been linked to enhanced cognitive performance (39), indicating a functionally advantageous state of improved cognitive flexibility. The current results further build up on earlier work positing a relationship between pupil size, the LC–NE system and task engagement (62). It is noteworthy that we observed correlations with transportation even using relatively short time-series. Future studies could look at the power law scaling exponents for pupil size fluctuation across longer time series, such as for whole chapters of novels (as in 53). We expect that the association between transportation and the LRTC scaling exponents should be even stronger when the immersed state lasts for a longer time.

Reduced blink count has been shown to be indicative of higher cognitive engagement (45, 46, 47) and self-reported interest in emotional stimuli (51). On the other hand, zoning-out or mind-wandering has been linked to higher blink count, indicating that increased blinking reflects drifting of attention away from the task (48). Spontaneous blinks have been associated with momentary inhibition of the dorsal attentional network that mediates the allocation of attention, and with activation of DMN (49), which has been implicated in mind-wandering or zoning-out (50). The present results thus provide evidence for a positive link between story transportation and cognitive engagement: higher transportation is related to higher engagement. However, future studies should include a measure of experienced cognitive load as a more concrete basis for the differentiation between cognitive and emotional influences on the pupillary signal.

In sum, the present study demonstrates that highly arousing horror texts are also more likely to induce transportation. During an immersive literary experience the cognitive capacities are highly focused on the text (e.g., 5), resulting in a state of improved cognitive flexibility characterized by fluency and increased cognitive engagement.

### Ethics and Conflict of Interest

The author(s) declare(s) that the contents of the article are in agreement with the ethics described in http://biblio.unibe.ch/portale/elibrary/BOP/jemr/ethics.html and that there is no conflict of interest regarding the publication of this paper.

### Acknowledgements

This research was conducted while Johanna K. Kaakinen was a senior research fellow at the Turku Institute for Advanced Studies. Jaana Simola was supported by the Academy of Finland (SA1294761), and a fellowship from the Helsinki Collegium for Advanced Studies.

## References

[b31] Alnæs, D., Sneve, M. H., Espeseth, T., Endestad, T., van de Pavert, S. H. P., & Laeng, B. (2014). Pupil size signals mental effort deployed during multiple object tracking and predicts brain activity in the dorsal attention network and the locus coeruleus. Journal of Vision (Charlottesville, Va.), 14(4), 1–20. 10.1167/14.4.1 1534-7362 24692319

[b54] Appel, M., Gnambs, T., Richter, T., & Green, M. C. (2015). The transportation scale–short form (TS–SF). Media Psychology, 18(2), 243–266. 10.1080/15213269.2014.987400 1521-3269

[b25] Aston-Jones, G., & Cohen, J. D. (2005). An integrative theory of locus coeruleus-norepinephrine function: Adaptive gain and optimal performance. Annual Review of Neuroscience, 28, 403–450. 10.1146/annurev.neuro.28.061604.135709 0147-006X 16022602

[b60] Bates, D., Maechler, M., Bolker, B., & Walker, S. (2015). Fitting Linear Mixed-Effects Models Using lme4. Journal of Statistical Software, 67(1), 1–48. 10.18637/jss.v067.i01 1548-7660

[b28] Beatty, J. (1982). Task-evoked pupillary responses, processing load, and the structure of processing resources. Psychological Bulletin, 91(2), 276–292. 10.1037/0033-2909.91.2.276 0033-2909 7071262

[b43] Beggs, J. M. (2007). The criticality hypothesis: how local cortical networks might optimize information processing. Philosophical Transactions of the Royal Society A: Mathematical, Physical and Engineering Sciences, 366(1864), 329-343. https://doi.org/10.1098/rsta.2007.2092 17673410

[b46] Bentivoglio, A. R., Bressman, S. B., Cassetta, E., Carretta, D., Tonali, P., & Albanese, A. (1997). Analysis of blink rate patterns in normal subjects. Movement Disorders, 12(6), 1028–1034. 10.1002/mds.870120629 0885-3185 9399231

[b21] Bradley, M. M., & Lang, P. J. (1994). Measuring emotion: The self-assessment manikin and the seman-tic differential. Journal of Behavior Therapy and Experimental Psychiatry, 25(1), 49–59. 10.1016/0005-7916(94)90063-9 0005-7916 7962581

[b22] Bradley, M. M., Miccoli, L., Escrig, M. A., & Lang, P. J. (2008). The pupil as a measure of emotional arousal and autonomic activation. Psychophysiology, 45(4), 602–607. 10.1111/j.1469-8986.2008.00654.x 0048-5772 18282202 PMC3612940

[b42] Chialvo, D. R. (2010). Emergent complex neural dynamics. Nature Physics, 6(10), 744–750. 10.1038/nphys1803 1745-2473

[b50] Christoff, K., Irving, Z. C., Fox, K. C. R., Spreng, R. N., & Andrews-Hanna, J. R. (2016). Mind-wandering as spontaneous thought: A dynamic framework. Nature Reviews. Neuroscience, 17(11), 718–731. 10.1038/nrn.2016.113 1471-003X 27654862

[b9] Citron, F. M. M. (2012). Neural correlates of written emotion word processing: A review of recent electrophysiological and hemodynamic neuroimaging studies. Brain and Language, 122(3), 211–226. 10.1016/j.bandl.2011.12.007 0093-934X 22277309

[b44] Deco, G., & Jirsa, V. K. (2012). Ongoing cortical activity at rest: Criticality, multistability, and ghost attractors. The Journal of Neuroscience : The Official Journal of the Society for Neuroscience, 32(10), 3366–3375. 10.1523/JNEUROSCI.2523-11.2012 0270-6474 22399758 PMC6621046

[b17] Faber, M., Mak, M., & Willems, R. (2020). Word skipping as an indicator of individual reading style during literary reading. Journal of Eye Movement Research, 13(3). Advance online publication. 10.16910/jemr.13.3.2 1995-8692 PMC7987350 33828800

[b56] Fink, L. K., Hurley, B. K., Geng, J. J., & Janata, P. (2018). A linear oscillator model predicts dynamic temporal attention and pupillary entrainment to rhythmic patterns. Journal of Eye Movement Research, 11(2), 12. 10.16910/jemr.11.2.12 1995-8692 PMC7898576 33828695

[b57] Franklin, M. S., Broadway, J. M., Mrazek, M. D., Smallwood, J., & Schooler, J. W. (2013). Window to the wandering mind: Pupillometry of spontaneous thought while reading. Quarterly Journal of Experimental Psychology, 66(12), 2289–2294. 10.1080/17470218.2013.858170 1747-0218 24313285

[b4] Gerrig, R. J. (1993). Experiencing narrative worlds: On the psychological activities of reading. Yale University Press.

[b38] Gilden, D. L., Thornton, T., & Mallon, M. W. (1995). 1/f noise in human cognition. Science, 267(5205), 1837–1839. 10.1126/science.7892611 0036-8075 7892611

[b37] Gilden, D. L. (2001). Cognitive emissions of 1/f noise. Psychological Review, 108(1), 33–56. 10.1037/0033-295X.108.1.33 0033-295X 11212631

[b5] Green, M. C., & Brock, T. C. (2000). The role of transportation in the persuasiveness of public narratives. Journal of Personality and Social Psychology, 79(5), 701–721. 10.1037/0022-3514.79.5.701 0022-3514 11079236

[b14] Hamann, S. (2001). Cognitive and neural mechanisms of emotional memory. Trends in Cognitive Sciences, 5(9), 394–400. 10.1016/S1364-6613(00)01707-1 1364-6613 11520704

[b58] Hardstone, R., Poil, S. S., Schiavone, G., Jansen, R., Nikulin, V. V., Mansvelder, H. D., & Linkenkaer-Hansen, K. (2012). Detrended fluctuation analysis: A scale-free view on neuronal oscillations. Frontiers in Physiology, 3, 450. 10.3389/fphys.2012.00450 1664-042X 23226132 PMC3510427

[b26] Hess, E. H., & Polt, J. M. (1960). Pupil size as related to interest value of visual stimuli. Science, 132(3423), 349–350. 10.1126/science.132.3423.349 0036-8075 14401489

[b29] Hess, E. H., & Polt, J. M. (1964). Pupil size in relation to mental activity during simple problem-solving. Science, 143, 1190–1192. 10.1126/science.143.3611.1190 0036-8075 17833905

[b47] Holland, M. K., & Tarlow, G. (1972). Blinking and mental load. Psychological Reports, 31(1), 119–127. 10.2466/pr0.1972.31.1.119 0033-2941 5055889

[b12] Hsu, C. T., Conrad, M., & Jacobs, A. M. (2014). Fiction feelings in Harry Potter: Haemodynamic response in the mid-cingulate cortex correlates with immersive reading experience. Neuroreport, 25(17), 1356–1361. 10.1097/WNR.0000000000000272 0959-4965 25304498

[b8] Jacobs, A. M. (2015). Neurocognitive poetics: Methods and models for investigating the neuronal and cognitive-affective bases of literature reception. Frontiers in Human Neuroscience, 9, 186. 10.3389/fnhum.2015.00186 1662-5161 25932010 PMC4399337

[b62] Jepma, M., & Nieuwenhuis, S. (2010). Pupil diameter predicts changes in the exploration-exploitation trade-off: Evidence for the adaptive gain theory. Journal of Cognitive Neuroscience, 23(7), 1587–1596. 10.1162/jocn.2010.21548 0898-929X 20666595

[b30] Kahneman, D., & Beatty, J. (1966). Pupil diameter and load on memory. Science, 154, 1583–1585. 10.1126/science.154.3756.1583 0036-8075 5924930

[b1] Kneepkens, E. W. E. M., & Zwaan, R. A. (1995). Emo-tions and literary text comprehension. Poetics, 23(1–2), 125–138. 10.1016/0304-422X(94)00021-W 0304-422X

[b6] Kuijpers, M. M., Hakemulder, F., Tan, E. S., & Doicaru, M. M. (2014). Exploring absorbing reading experiences: Developing and validating a self-report scale to measure story world absorption. Scientific Study of Literature, 4(1), 89–122. 10.1075/ssol.4.1.05kui 2210-4372

[b15] Lang, P. J., Greenwald, M. K., Bradley, M. M., & Hamm, A. O. (1993). Looking at pictures: Affective, facial, visceral, and behavioral reactions. Psychophysiology, 30(3), 261–273. 10.1111/j.1469-8986.1993.tb03352.x 0048-5772 8497555

[b40] Linkenkaer-Hansen, K., Nikouline, V. V., Palva, J. M., & Ilmoniemi, R. J. (2001). Long-range temporal correlations and scaling behavior in human brain oscillations. The Journal of Neuroscience : The Official Journal of the Society for Neuroscience, 21(4), 1370–1377. 10.1523/JNEUROSCI.21-04-01370.2001 0270-6474 11160408 PMC6762238

[b51] Maffei, A., & Angrilli, A. (2019). Spontaneous blink rate as an index of attention and emotion during film clips viewing. Physiology & Behavior, 204, 256–263. 10.1016/j.physbeh.2019.02.037 0031-9384 30822434

[b18] Magyari, L., Mangen, A., Kuzmičová, A., Jacobs, A., & Lüdtke, J. (2020). Eye movements and mental im-agery during reading of literary texts with different narrative styles. Journal of Eye Movement Research, 13(3). Advance online publication. 10.16910/jemr.13.3.3 1995-8692 PMC7886417 33828798

[b20] Mauss, I. B., & Robinson, M. D. (2009). Measures of emotion: A review. Cognition and Emotion, 23(2), 209–237. 10.1080/02699930802204677 0269-9931 19809584 PMC2756702

[b35] Mesin, L., Monaco, A., &Cattaneo, R. (2013). Inves-tigation of nonlinear pupil dynamics by recurrence quantification analysis. BioMed research international, 2013(420509). https://doi.org/10.1155/2013/420509 PMC3804145 24187665

[b2] Miall, D. S., & Kuiken, D. (2002). A feeling for fiction: Becoming what we behold. Poetics, 30(4), 221–241. 10.1016/S0304-422X(02)00011-6 0304-422X

[b49] Nakano, T., Kato, M., Morito, Y., Itoi, S., & Kitazawa, S. (2013). Blink-related momentary activation of the default mode network while viewing videos. Pro-ceedings of the National Academy of Sciences, 110(2), 702–706. 10.1073/pnas.1214804110 1091-6490 23267078 PMC3545766

[b10] Nummenmaa, L., Saarimäki, H., Glerean, E., Gotsopoulos, A., Jääskeläinen, I. P., Hari, R., & Sams, M. (2014). Emotional speech synchronizes brains across listeners and engages large-scale dynamic brain networks. NeuroImage, 102, 498–509. 10.1016/j.neuroimage.2014.07.063 1053-8119 25128711 PMC4229500

[b11] Nummenmaa, L., & Saarimäki, H. (2019). Emotions as discrete patterns of systemic activity. Neuroscience Letters, 693, 3–8. 10.1016/j.neulet.2017.07.012 0304-3940 28705730

[b3] Oatley, K. (1995). A taxonomy of the emotions of literary response and a theory of identification in fic-tional narrative. Poetics, 23(1–2), 53–74. 10.1016/0304-422X(94)P4296-S 0304-422X

[b41] Palva, S., & Palva, J. M. (2018). Roles of brain criti-cality and multiscale oscillations in temporal predic-tions for sensorimotor processing. Trends in Neurosciences, 41(10), 729–743. 10.1016/j.tins.2018.08.008 0166-2236 30274607

[b27] Partala, T., & Surakka, V. (2003). Pupil size variation as an indication of affective processing. International Journal of Human-Computer Studies, 59(1-2), 185–198. 10.1016/S1071-5819(03)00017-X 1071-5819

[b59] Peng, C.-K., Havlin, S., Stanley, H. E., & Goldberger, A. L. (1995). Quantification of scaling exponents and crossover phenomena in nonstationary heartbeat time series. Chaos (Woodbury, N.Y.), 5, 82–87. 10.1063/1.166141 1054-1500 11538314

[b13] Posner, J., Russell, J. A., & Peterson, B. S. (2005). The circumplex model of affect: An integrative approach to affective neuroscience, cognitive development, and psychopathology. Development and Psychopathology, 17(3), 715–734. Advance online publication. 10.1017/S0954579405050340 0954-5794 16262989 PMC2367156

[b61] R Core Team (2018). R: A language and environment for statistical computing. R Foundation for Statistical Computing, Vienna, Austria. URL https://www.R-project.org/

[b53] Riese, K., Bayer, M., Lauer, G., & Schacht, A. (2014). In the eye of the recipient: Pupillary responses to suspense in literary classics. Scientific Study of Literature, 4(2), 211–232. 10.1075/ssol.4.2.05rie 2210-4372

[b7] Ryan, M.-L. (2001). Narrative as virtual reality: Im-mersion and interactivity in literature and electronic media. Johns Hopkins University Press.

[b39] Simola, J., Zhigalov, A., Morales-Muñoz, I., Palva, J. M., & Palva, S. (2017). Critical dynamics of endogenous fluctuations predict cognitive flexibility in the Go/NoGo task. Scientific Reports, 7(1), 2909. 10.1038/s41598-017-02750-9 2045-2322 28588303 PMC5460255

[b48] Smilek, D., Carriere, J. S. A., & Cheyne, J. A. (2010). Out of mind, out of sight: Eye blinking as indicator and embodiment of mind wandering. Psychological Science, 21(6), 786–789. 10.1177/0956797610368063 0956-7976 20554601

[b55] SR Research (2017). EyeLink® 1000 Plus Technical Specifications. https://www.sr-research.com/wp-content/uploads/2017/11/eyelink-1000-plus-specifications.pdf

[b24] Steinhauer, S. R., Siegle, G. J., Condray, R., & Pless, M. (2004). Sympathetic and parasympathetic innervation of pupillary dilation during sustained processing. International Journal of Psychophysiology, 52(1), 77–86. 10.1016/j.ijpsycho.2003.12.005 0167-8760 15003374

[b45] Stern, J. A., Boyer, D., & Schroeder, D. (1994). Blink rate: A possible measure of fatigue. Human Factors, 36(2), 285–297. 10.1177/001872089403600209 0018-7208 8070793

[b32] Unsworth, N., & Robison, M. K. (2017). A locus coeruleus-norepinephrine account of individual differences in working memory capacity and attention control. Psychonomic Bulletin & Review, 24(4), 1282–1311. 10.3758/s13423-016-1220-5 1069-9384 28108977

[b36] Usui, S., & Stark, L. (1982). A model for nonlinear stochastic behavior of the pupil. Biological Cybernetics, 45(1), 13–21. 10.1007/BF00387209 0340-1200 7126688

[b52] Wallentin, M., Nielsen, A. H., Vuust, P., Dohn, A., Roepstorff, A., & Lund, T. E. (2011). Amygdala and heart rate variability responses from listening to emotionally intense parts of a story. NeuroImage, 58(3), 963–973. 10.1016/j.neuroimage.2011.06.077 1053-8119 21749924

[b23] Wang, C.-A., Baird, T., Huang, J., Coutinho, J. D., Brien, D. C., & Munoz, D. P. (2018). Arousal Effects on Pupil Size, Heart Rate, and Skin Conductance in an Emotional Face Task. Frontiers in Neurology, 9, 1029. 10.3389/fneur.2018.01029 1664-2295 30559707 PMC6287044

[b33] van den Brink, R. L., Murphy, P. R., & Nieuwenhuis, S. (2016). Pupil diameter tracks lapses of attention. PLoS One, 11(10), e0165274. 10.1371/journal.pone.0165274 1932-6203 27768778 PMC5074493

[b16] Vogt, J., De Houwer, J., Koster, E. H., Van Damme, S., & Crombez, G. (2008). Allocation of spatial attention to emotional stimuli depends upon arousal and not valence. Emotion (Washington, D.C.), 8(6), 880–885. https://doi.ord/10.1037/a0013981 10.1037/a0013981 1528-3542 19102600

[b19] Xue, S., Lüdtke, J., Sylvester, T., & Jacobs, A. (2019). Reading Shakespeare sonnets: Combining quantitative narrative analysis and predictive model-ing - an eye tracking study. Journal of Eye Movement Research, 12(5). Advance online publication. 10.16910/jemr.12.5.2 1995-8692 PMC7968390 33828746

[b34] Zokaei, N., Board, A. G., Manohar, S. G., & Nobre, A. C. (2019). Modulation of the pupillary response by the content of visual working memory. Proceedings of the National Academy of Sciences of the United States of America, 116(45), 22802–22810. 10.1073/pnas.1909959116 0027-8424 31636213 PMC6842592

